# Effects of exercise training on nigrostriatal neuroprotection in Parkinson’s disease: a systematic review

**DOI:** 10.3389/fnins.2024.1464168

**Published:** 2025-01-08

**Authors:** Shahid Ishaq, Iqbal Ali Shah, Shin-Da Lee, Bor-Tsang Wu

**Affiliations:** ^1^PhD Program in Healthcare Science, College of Healthcare Science, China Medical University, Taichung, Taiwan; ^2^Department of Physical Therapy, China Medical University, Taichung, Taiwan; ^3^Department of Senior Citizen Service Management, National Taichung University of Science and Technology, Taichung, Taiwan

**Keywords:** exercise training, neuroprotection, nigrostriatum, Parkinson’s disease, treadmill training

## Abstract

**Introduction:**

Parkinson’s disease (PD) is characterized by progressive neurodegeneration within the nigrostriatum, leading to motor dysfunction. This systematic review aimed to summarize the effects of various exercise training regimens on protein or gene expression within the nigrostriatum and their role in neuroprotection and motor function improvement in animal models of Parkinson’s disease (PD).

**Methods:**

PubMed, EMBASE, and Web of Science were searched up to June 2024 and included sixteen studies that adhere to PRISMA guidelines and CAMARADES checklist scores ranging from 4 to 6 out of 10. Various exercise training regimens, administered 5 days per week for 6.5 weeks, were applied to MPTP, 6-OHDA, and PFF-α-synuclein-induced PD animal models.

**Results:**

Exercise training was found to downregulate the inflammatory pathway by attenuating α-synuclein aggregation, inhibiting the TLR/MyD88/IκBα signaling cascade and NF-κB phosphorylation, and decreasing pro-inflammatory cytokines IL-1β and TNF-α while increasing anti-inflammatory cytokines IL-10 and TGF-β within the nigrostriatum. It also inhibited the ASC and NLRP3 inflammasome complex and reduced the BAX/ Bcl-2 ratio and caspase-1/3 proteins, thereby decreasing neuronal apoptosis in the nigrostriatum. Exercise training elevated the expression of Pro-BDNF, BDNF, GDNF, TrkB, and Erk1/2, providing neurotrophic support to dopaminergic neurons. Furthermore, it upregulated the dopaminergic signaling pathway by increasing the expression of TH, DAT, PSD-95, and synaptophysin in the nigrostriatum.

**Discussion:**

The findings suggested that exercise training downregulated inflammatory and apoptotic pathways while upregulated BDNF/GDNF pathways and dopaminergic signaling within the nigrostriatum. These molecular changes contributed to neuroprotection, reduced dopaminergic neuron loss, and improved motor function in PD animal models.

**Systematic review registration:**

CRD42024484537 https://www.crd.york.ac.uk/prospero/#recordDetails.

## 1 Introduction

Parkinson’s disease (PD) is the second most common neurodegenerative disease, affecting approximately 1–2% of the elderly population (Jang et al., 2017). It is characterized by the progressive degeneration of dopaminergic neurons in the substantia nigra, reduced dopamine levels in the striatum, and impaired synaptic activity (Shi et al., 2019). Clinically, PD manifests as resting tremors, rigidity, bradykinesia, postural instability, and motor dysfunction (Tajiri et al., 2010). Motor symptoms of PD include increased immobility, poor motor coordination, balance issues, akinesia, and reduced walking, which are accompanied by altered gait parameters such as reduced stride length, increased stride time, decreased stride frequency, and abnormal foot placement angles (Chen et al., 2018; Wang et al., 2021; Leem et al., 2023).

The pathophysiology of PD involves several molecular mechanisms, including the accumulation of *α*-synuclein-containing Lewy bodies, neuroinflammation, and neuronal apoptosis, all of which contribute to the degeneration of the nigrostriatal pathway (Brys et al., 2017; Schulz-Schaeffer, 2010). The loss of dopaminergic neurons in PD triggers a cascade of molecular events, such as impaired BDNF/GDNF neurotrophic support and exacerbated synaptic dysfunction in dopaminergic neurons within the nigrostriatal pathway, ultimately leading to motor impairment (Palasz et al., 2019b). Exercise training is noninvasive, and nonpharmacological therapeutic intervention has to be proven beneficial for improving motor and cognitive function and reducing the symptomatic severity in clinical studies (Petzinger et al., 2013; Ahlskog, 2018). Exercise training has been reported to attenuate neuronal inflammation, and dopaminergic neuron loss in the nigrostriatum and enhance synaptic activity in PD animal models, contributing to motor function restoration (Shi et al., 2017; Tajiri et al., 2010).

Nigrostriatal neuronal inflammation in PD increases due to the accumulation of the Lewy body primarily the *α*-Synuclein (α-syn) in dopaminergic neurons, which triggers toll-like receptor (TLR) activation (Koo et al., 2017b). This activation initiates downstream signaling through myeloid differentiation primary response 88 (MYD88), tumor necrosis factor receptor-associated factor 6 (TRAF6), TGF-beta activated kinase 1 (TAK-1), and inhibitor of kappa B alpha (IκBα) (Jang et al., 2017). This upregulation promotes the phosphorylation of Nuclear Factor kappa B (NF-κB), increasing pro-inflammatory cytokines such as interleukin 1 beta (IL-1*β*) and tumor necrosis factor-alpha (TNF-α), and downregulation of anti-inflammatory cytokines interleukin 10 (IL-10) and transforming growth factor beta (TGF-β) leading to inflammation of nigrostriatal dopaminergic neurons (Wang et al., 2021; Leem et al., 2023). The reduced expression of bone marrow tyrosine kinase in chromosome X (BMX) protein in the PD striatum fails to maintain the inflammatory balance(Hsueh et al., 2018). The increased expression of ionized microglia activating protein calcium-binding adapter molecule 1 (Iba-1) and astrocytes activating protein Glial Fibrillary Acidic Protein (GFAP) increase the nigrostriatal neuronal inflammation in PD brain (Real et al., 2017).

Neuronal apoptosis is another molecular process driven by PD pathology, the damage-associated molecular patterns (DAMPs) upregulate pro-apoptotic factors such as caspase-1, caspase-3, cleaved caspase-3, and Bcl-2 Associated X Protein (BAX), while simultaneously downregulating the anti-apoptotic protein Bcl-2, resulting in the progressive loss of dopaminergic neurons (Koo et al., 2017b). The increase in cathepsin D, responsible for breaking down essential cellular components within the NS, further contributes to neuronal degeneration and death (Leem et al., 2023). Moreover, the activation of the NOD-like receptor family, pyrin domain containing 3 (NLRP3), and the ASC complex in response to cellular stress exacerbates neuroinflammation and apoptosis in the NS of the PD brain (Wang et al., 2021).

Brain-derived neurotrophic factor (BDNF) and glial cell line-derived neurotrophic factor (GDNF) play a crucial role in maintaining synaptic plasticity in dopaminergic neurons in the striatum of the PD brain (Malczynska-Sims et al., 2020). However, PD disrupts the processing of pro-BDNF to mature BDNF, impairing the BDNF–TrkB signaling pathway, which is crucial for dendritic growth, spine maturation, and synaptic plasticity through dopamine D1 receptor-dependent cyclic AMP (cAMP) signaling in the NS (Palasz et al., 2020). This neurotrophic support to the dopaminergic neurons facilitates the synaptic plasticity in the NS of PD brain (Chang and Wang, 2021).

Dopaminergic signaling, which is essential for motor function, is severely compromised in PD due to the downregulation of tyrosine hydroxylase (TH). This downregulation inhibits the conversion of tyrosine into L-dihydroxyphenylalanine (L-DOPA), the precursor for dopamine synthesis, exacerbating the pathogenesis of PD and impairing synaptic plasticity in the nigrostriatal medium spiny neurons (Kim et al., 2014). Additionally, the decreased expression of synaptic proteins such as synaptophysin and postsynaptic density protein 95 (PSD-95) indicates the structural synaptic changes that contribute to the dysfunction in the NS of PD animal model (Toy et al., 2014).

Recently, exercise training has emerged as a promising approach for modulating inflammatory, apoptotic, BDNF/GDNF, and dopaminergic signaling pathways, thereby offering neuroprotection in the NS of PD animal models (da Silva et al., 2016; Ferreira et al., 2021; Leem et al., 2023). However, some studies report conflicting outcomes, suggesting that while exercise training may improve motor function, it might not uniformly affect nigrostriatal neuroprotection in PD models (Hood et al., 2016; Churchill et al., 2017). We plan to address the effects of various exercise training on the molecular pathways implicated in the nigrostriatum of PD animal models. This systematic review aimed to summarize the effects of different exercise training programs on the regulation of protein or gene expressions in inflammatory, apoptotic, BDNF/GDNF, and dopaminergic signaling pathways, and their contribution to nigrostriatal neuronal protection and motor function recovery in various animal models of Parkinson’s disease.

## 2 Methodology

### 2.1 Study design

This systematic review included controlled-trial animal studies with separate exercise training groups and well-defined control groups. Studies published in English, without restriction on the publication date, were included. Only animal PD models, including both male and female subjects of any species, that measured gene or protein expressions within the nigrostriatum were included. Additionally, this study included bilaterally induced PD animal models to evaluate the effect of exercise training on motor function. The exclusion criteria encompassed articles with inappropriate study design, crossover studies, case reports, conference papers, abstracts, and review articles. Studies were excluded if they did not provide sufficient information about the animal species and the induction of the PD model. Exercise training of various types and parameters, with clear information about the duration of treatment, was included. Animals not exposed to exercise intervention, exposed only to one or two sessions, or parallelly treated with any other intervention were excluded. The sedentary PD models, not subjected to exercise training or any other intervention, were used as the comparator. The primary objective of this systematic review was to record the protein expression within the nigrostriatum of the PD brain regulated by exercise training involving different pathways, including the inflammatory pathway, apoptotic pathway, BDNF/GDNF-related pathway, and dopaminergic signaling pathway. The secondary objective was to summarize the effect of exercise training on motor function in PD animal models.

### 2.2 Search strategy

PubMed, Web of Science, and EMBASE databases were searched until June 2024, using the following search string: (Exercise training OR physical activity OR aerobics exercise OR treadmill exercise) AND (Parkinson OR Parkinson’s disease) AND (neuroprotection OR dopamine system OR dopamine neuron OR synaptic plasticity OR TH neurons) AND (substantia nigra OR striatum) AND (animal model) to retrieve relevant papers. After duplicate removal titles, abstracts and full-text screening were carried out, respectively, to find the most appropriate studies for this systematic review. Additionally, references from the included studies were reviewed to identify other relevant papers. The process of study selection was independently conducted by two reviewers and disagreement was discussed with a third reviewer to make the final decision.

### 2.3 Data collection

Two independent reviewers extracted the data, including study characteristics and outcomes, by scrutinizing the included studies’ figures, tables, graphs, and text. For study characteristics, we extracted data such as the first author’s name, publication year, PD model details (sample size, species, gender, age, and weight), and exercise protocol specifics (speed, frequency, duration, and total period). we recorded protein expression regulated by exercise training involving the inflammatory pathway, apoptotic pathway, BDNF/GDNF-related pathway, and dopaminergic signaling pathway in the nigrostriatum of the PD brain as well as motor function.

### 2.4 Study quality

The study quality was assessed using the CAMARADES checklist (Auboire et al., 2018), which consists of ten items. Two authors independently evaluated and completed the pre-designed datasheets of the CAMARADES checklist. Any disagreements were resolved through discussion among the two evaluators and the involvement of a third consultant.

### 2.5 Data synthesis

The results of the search strategies were depicted in the PRISMA flowchart (Page et al., 2021) for reporting this systematic review. Text and tables were used to present the study characteristics and outcomes. Summaries of the different PD animal models, exercise training protocols, and types of outcomes were provided for the study characteristics. For the outcomes, the effects of exercise training on nigrostriatal neuronal protection were highlighted, focusing on protein expression involved in the downregulation of inflammatory and apoptotic pathways, upregulation of BDNF/GDNF-related and dopaminergic signaling pathways, and improvements in motor function. This synthesized data was used to draw the hypothetical pathways.

## 3 Results

**Figure 1:**
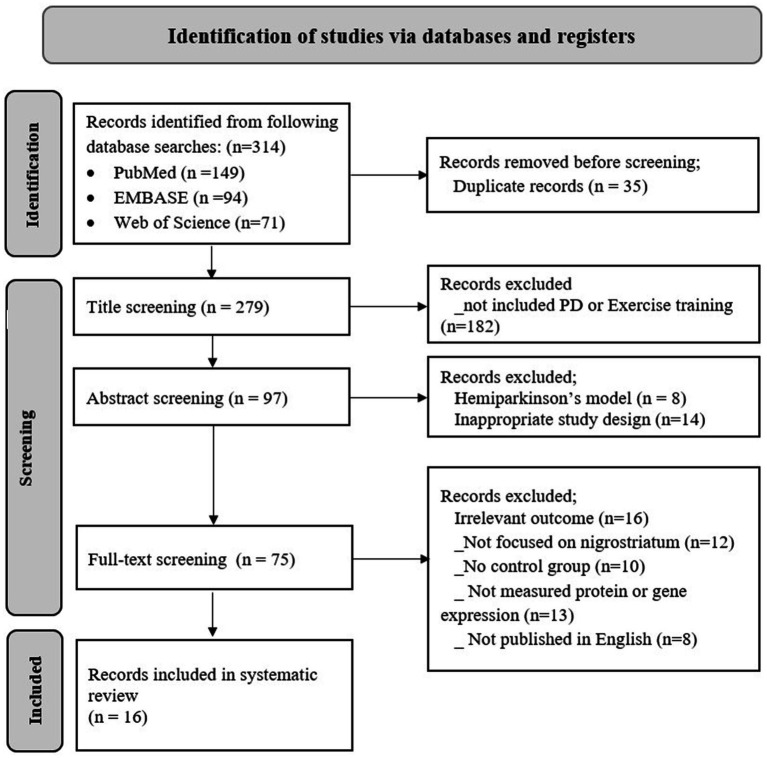
PRISMA flow diagram for selection methodology.

A total of 314 articles were identified across PubMed (*n* = 149), EMBASE (*n* = 94), and Web of Science (*n* = 71) (Figure 1). After removing 35 duplicates, 279 articles remained. During title screening, 182 studies were excluded as they did not involve Parkinson’s disease (PD) models or exercise training. Eight studies using hemiparkinsonian models and 14 studies with inappropriate designs, such as cohort studies, case reports, or crossover studies, were excluded during abstract screening. Subsequently, in the full-text screening, 59 studies did not meet the eligibility criteria: 16 did not measure relevant outcomes, 12 were not focused on brain location nigrostriatum, 10 did not include the control group, 13 did not list protein or gene expressions, and 8 were not published in English. Finally, 16 studies involved different PD animal models and investigated protein or gene expression regulated by exercise training in the nigrostriatum.

The studies included different PD animal models such as the 1-methyl-4-phenyl-1,2,3,6-tetrahydropyridine (MPTP) model (*n* = 11), the 6-hydroxydopamine (6-OHDA) model (*n* = 4), and the *α*-syn-PFFs model (*n* = 1). Among these studies (*n* = 15), males were predominantly used, with only one study (*n* = 1) including female Sprague rat animals. Additionally, (*n* = 13) studies included mice, while (*n* = 3) included rats. The animals were mainly young, falling in the age range of 7–12 weeks, except for one study which included 64-week-old mice, and two studies which did not disclose the age of the animals.

The effects of the heterogeneous exercise training protocol on motor functions and protein expressions involving the inflammatory pathways, apoptotic pathway, BDNF/GDNF-related pathway, and dopaminergic signaling pathway are summarized in (Table 1). Various exercise training regimens were employed in the studies, including treadmill training, rotarod walking exercise, and running wheel activity. Treadmill training protocols included different regimens for 4 to 10 weeks with speeds varying from 3 to 18 m/min and durations from 30 to 60 min/day, while only one treadmill training protocol lasted for 18 weeks. Rotarod walking exercise involves forced training for 4 weeks at speeds of 8–20 rpm and durations of 20–38 min/day. Additionally, voluntary running wheel exercise spans 6 weeks. These exercises are conducted 3 to 5 days per week, with some protocols incorporating progressive speed increments, while most programs emphasize consistent frequency and duration to observe their effects on nigrostriatal neuronal protection in the PD animal models (Table 1).

**Table 1:** Effects of exercise training on nigrostriatal dopaminergic neuronal protection.

Study	Sample	Disease Model	Involved Brain region	Exercise type	Exercise parameters	Motor Function	Inflammatory Pathway	Apoptotic Pathway	BDNF/GDNF Pathway	Dopaminergic Signaling Pathway	Others
Marino et al. (2023)	S: Male Wister rats (*n* = 123). A: 8–12 weeks W: 300–350 g	α-syn-PFFs.	VTA, SNpc & Striatum	Treadmill training (Forced)	Sp: progressive 3-11 m/min D: 30 min/day F: 5 days/week P:4 weeks habituation and 4 weeks forced training	PA: latency↑, immobility time↑ PA + Ex: latency↓, immobility↓ (45^o^ inclined grid walking)	PA: P-α Synclein↑ PA + Ex: P-α Synclein↓,		PA: BDNF↓ PA + Ex: BDNF↑, TrkB Ө,	PA: TH↓, DAT↓, PA + Ex: TH↑, DAT↑,	
Leem et al. (2023)	S: Male mice (*n* = 72–80) A: 7 weeks	MPTP	SNpc & striatum	Rota rod walking exercise (Forced)	Sp:8–20 rpm, D: 20–38 min/day, F: 5 days/week P: 4 weeks	PA: motor coordination↓, Akinesia↑, retention time on rod↓, PA + Ex: motor coordination improved, Akinesia↓, retention time on rod↑, (Rota-rod test)	PA: pGSK3β (Y216)↑, pGSK3β (S9)↓, pGSK3β↓, MMP-3↑, −α Synclein↑, P-α Synclein↑ p-IκBα↑, t-IκBα↑, NF-kB↑, iNOS↑, IL-1β↑, TNF-α↑, IL-10↓ & TGF-β↓, PA + Ex: pGSK3β (Y216)↓ pGSK3β (S9)↑, pGSK3β↑, MMP-3↓, α Synclein↓, P-α Synclein↓, p-IκBα↑, t-IκBα↓, NF-kB↓, iNOS↓, IL-1β↓, TNF-α↓, IL-10↑ & TGF-β↑,	PA: cathepsin D↑, PA + Ex: cathepsin D↓,	PA: BDNF↓, GDNF↓, PA + Ex: BDNF↑, GDNF↑,	PA: TH↓, PA + Ex: TH↑,	PA: 4-HNE↑, Nrf2↓, HO-1↓, NQO1, PGC1-α↓, Nurr1↓, & p-AMPK↓ PA + Ex: 4-HNE↓, Nrf2↑, HO-1↑, NQO1, PGC1-α↑, Nurr1↑, & p-AMPK↑
Wang et al. (2022)	S: Male mice (n 54) A; W: 25 ± 2 g,	6 OHDA	SNpc Striatum	Treadmill training	Sp:12–18 m/min, D: 40 min/day F: 5 days/week P:4 weeks	PA: Distance traveled↓ (OFT), Retention time↓ (Rota-rod test), Stride length↓ PA + Ex: Distance traveled↑ (OFT), Retention time↑ (Rota-rod test), Stride length↑			PA: Erk1/2↑, MAPK-p-Erk1/2↑, PA + Ex: Erk1/2↓, MAPK-p-Erk1/2↓	PA: TH↓, PA + Ex: TH↑,	
Wang et al. (2021)	S: Male mice A: 8 weeks W: 20–30 g	MPTP	SN & striatum	Treadmill training	Sp:12 m/min, D: 60 min/day F: 5 days/week P:6 weeks	PA: Distance traveled↓, equilibrium impaired (OFT) Time of t-turn & t-total↓(Pole test) PA + Ex: Distance traveled↑, equilibrium improved (OFT) Time of t-turn & t-total↑(Pole test)	PA: Pro-IL-Iβ↑, IL-1B↑, TLR4↑, MyD88↑, IκBα/ p-IκBα↑, P-65↑, NF-κB↑, Iba-1↑ PA + Ex: Pro-IL-Iβ↓, IL-1B↓, TLR4↓, MyD88↓, IκBα/ p-IκBα↓, P-65↓, NF-κB↓, Iba-1↓	PA: NLRP3↑, ASC↑, Pro-caspase1↑, caspase1↑, BAX/Bcl-2↑ PA + Ex: NLRP3↓, ASC↓, Pro-caspase↓, caspase↓, BAX/Bcl-2↓		PA: TH↓, DA↓ PA + Ex: TH↑, DA↑	
Palasz et al. (2019a)	S: Male mice (*n* = 67) A: 12 weeks	MPTP	SNpc, Striatum, VTA & mid brain	Treadmill exercise	Sp:15 m/min, D: 40 min/day, F: 5 days/week P: 10 weeks	PA: Distance traveled↓, (OFT) PA + Ex: Distance traveled↑, (OFT)	PA: CD11b↑, Iba1↑, GFAP↑, IL-1β↑ PA + Ex: CD11b↓, Iba1↓, GFAP↓, IL-1β↓		PA: BDNF↓, GDNF↓ PA + Ex: BDNF↑, GDNF↑	PA: TH↓, PA + Ex: TH↑,	
Hsueh et al. (2018)	S: female rats. (*n* = 71) A: 8–12 weeks W: 200–300 g	6 OHDA	SNpc & striatum	Running wheel (voluntary)	Sp: 6 weeks training 16.524 ± 0.9864 m/min. P: 6 weeks	PA: FST-immobility↑, Rt.↑, Balance↓, Walking speed↓, Stride length↓ PA + Ex: immobility↓, Rt.↓, Balance improved, Walking speed↑, Stride length↑	PA: BMX↓, PA + Ex: BMX↑,		PA: BDNF↓, PA + Ex: BDNF↑,	PA: TH↓, PA + Ex: TH↑,	
Real et al. (2017)	S: Male rats (*n* = 92) A: 12 weeks	6 OHDA	SNpc & Striatum	Treadmill training	Sp: 10 m/min D: 40 min/day F: 3 days/week P: 4 weeks	PA: Rotational Asymatry↑, PA + Ex: Rotational A symmetry↓	PA: CD-11c/b↑, GFAP↑, iNOS↑, PA + Ex: CD-11c/b↓, GFAP↓, iNOS↓,			PA: TH↓, PA + Ex: TH↑	
Koo et al. (2017b)	S: Male mice (*n* = 30) A: 7 weeks	MPTP	SNpc and striatum	Treadmill training	Sp:10 m/min D: 60 min/day F: 5 days/ week P:8 weeks	PA: motor coordination↓, retention time↓ PA + Ex: motor coordination improved, retention time↑ (Rota-rod test)	PA: α- Synuclein↑, TLR2↑, MYD88↑, TRAF6↑, P-TAK-1/T-TAK1↑, p-IκBα/t-IκBα↑, NF-kB↑, P- NF-kB↑, TNF-α↑, IL-1β↑, Iba-1↑, PA + Ex: α- Synuclein↓, TLR2↓, MYD88↓, TRAF6↓, P-TAK-1↓, T-TAK-1↓, p-IκBα/t-IκBα↓, NF-kB↓, P- NF-kB↓, TNF-α↓, IL-1β↓, Iba-1↓	PA: caspase 3↑, cleaved caspase3↑,Bcl-2↓, NADPH(p47phox↑, gp91phox↑, p22phox↑) PA + Ex: caspase 3↑, cleaved caspase-↑, Bcl-2↑, NADPH(p47phox↓, gp91phox↓, p22phox↓)		PA: TH↓, DAT↓ PA + Ex: TH↑, DAT↑	
Jang et al. (2017)	S: Male mice (*n* = 30) A: 8 weeks	MPTP	SN & striatum	Treadmill training	Sp: 10 m/min D: 60 min/day F: 5 days/week P: 8 weeks	PA: motor coordination↓, retention time↓ PA + Ex: motor coordination improved, retention time↑ (Rota-rod test)	PA: α- Synuclain↑, TLR2↑, MYD88↑, TRAF6↑, P-TAK-1↑, T-TAK-1↑, p-IκBα/t-IκBα↑, P-NF-kB↑, TNF-α↑, IL-1β↑, PA + Ex: α- Synuclain↓, TLR2↓, MYD88↓, TRAF6↓, P-TAK-1↓, T-TAK-1↓, p-IκBα/t-IκBα↓, NF-kB↓, TNF-α↓, IL-1β↓,			PA: TH↓ PA + Ex: TH↑	
Churchill et al. (2017)	S: Male mice (*n* = 69) A: 8 weeks	MPTP	SNpc & striatum	Treadmill training	Sp: 11 m/min D: 60 m/day F: 5 days/ week P: 4 weeks	PA: stride time↓, stride length↓, stride frequency↓, foot placement angle ↑ PA + Ex: stride time Ө stride length ᴓ stride frequency↑ foot placement angle ↓	In SN PA: Iba-1↑ PA + Ex: Iba-1Ө In Striatum PA: Iba-1↑ PA + Ex: Iba-1↓		PA: BDNF↓, PA + Ex: BDNFӨ,	PA: TH↓, DAT↑ PA + Ex: THӨ, DATӨ,	PA: GLT-1↓ PA + Ex: GLT-1↑
Shin et al. (2016)	S: Male mice (*n* = 40) A: 10 weeks W: 25 ± 3 g	MPTP	SNpc & Striatum	Treadmill training	Sp: 12 m/min, D: 30 min/day, F: 5 days/week P: 4 weeks	PA: fall↑, walking speed↓, motor coordination↓ PA + Ex: fall↓, walking speed ↑, coordination improved (Rota-rod test)				PA: TH↓, Syp↓, PSD-95↓, PA + Ex:TH↑, Syp.↑, PSD-95↑,	
Hood et al. (2016)	S: Male mice (*n* = 34) A: 64 weeks,	MPTP	SN & striatum	Treadmill trainig	Sp: 11 m/min, D: 60 min/day, F: 5 days/week P: 4 weeks	PA: Parallel rod activity↓, Bean Breaks↓ PA + Ex: Parallel rod activity Improved, Bean Breaks↑				PA: TH↓, DAT↓, DA↓ PA + Ex: THӨ, DATӨ, DAӨ	
Toy et al. (2014)	S: Male mice (*n* = 64) A: 8–10 weeks	MPTP	SN & Striatum and Basal ganglia	Intensive Treadmill exercise	Sp: Progressive 10–24 m/min D: F: 5 days/week P: 6 weeks	PA: running speed↓ PA + Ex: Restore running speed				PA: Syp Ө, PSD-95↓, PA + Ex: Syp.↑, PSD-95↑,	
Tuon et al. (2014)	S: Male mice (*n* = 72) A: Adult W: 25–30 g	6 OHDA	SN, Striatum and Hippocampus	Treadmill training	Sp: Progressive 13-17 m/ min D: 50 min/day F: 3–4 days/weeks P: 8 weeks	PA: FST- Immobility↑, OFT-t-turn↑ PA + Ex: Immobility↓, OFT-t-turn↓			PA: Pro-BDNF↓, BDNF↓ & Tr-kB↓ PA + Ex: Pro-BDNF↑, BDNF↑ & Tr-kB↑		
Lau et al. (2011)	S: Male mice (*n* = 135) A: 6–10 weeks	MPTP + Probenoid	SN & striatum	Treadmill training	Sp: Progressive 15 m/min D: 40 min/day F: 5 days/week P:18 weeks	PA: Latency↑, balance↓, motor coordination↓ PA + Ex: latency↓, balance improved, coordination improved (Balance beam)			PA: BDNF↓, GDNF↓ PA + Ex: BDNF↑, GDNF↑	PA: TH↓, DAT↓ PA + Ex: TH↑, DAT↑	PA: Mn-SOD↓, Cu-Zn SOD↓ PA + Ex: Mn-SOD↑, Cu-Zn SOD↑
Smith et al. (2011)	S: Male mice (*n* = 40) A: 8–10 weeks	MPTP	SNpc & striatum	Treadmill trainnig	Sp: 11 m/min D: 60 min/day F: 5 days/weeks P: 4 weeks	PA: Poor parallel rod activity PA + Ex: Improved parallel rod acticity				PA: TH↓, DAT↓ PA + Ex: TH↑, DAT↑	

This table summarizes the effects of exercise training (Ex) on various animal models of Parkinson’s disease (PD); 1-methyl-4-phenyl-1,2,3,6-tetrahydropyridine (MPTP), 6-hydroxydopamine (6-OHDA), and perforated fibrils (PFF). Abbreviations used: Subject (S), sample size (n), age (A), weight (W), gram (g), substantia nigra (SN), and substantia nigra pars compacta (SNpc). The Ex parameters are speed (Sp), meter /minutes (m/min), time duration (D), frequency (F), and period (P). Parkinson’s group (PA), Parkinson’s and Exercise group (PA + Ex), open field test (OFT), forced swimming test (FST), total (t), phosphorylation (p), decreased/ downregulation (↓), increased/up-regulation (↑), no significant effect (Ө), no effect (ᴓ). Matrix metalloproteinase-3 (MMP-3), toll-like receptor (TLR) & binding protein myeloid differentiation primary response 88 (MYD88), bone marrow tyrosine kinase in chromosome X (BMX) protein, tumor necrosis factor receptor-associated factor 6 (TRAF6), TGF-beta activated kinase 1 (TAK-1), an inhibitor of kappa B alpha (IκBα), nuclear factor kappa B (NF-kB), inducible nitric oxide synthase (iNOS), interleukin 1 beta (IL-1β), tumor necrosis factor-alpha (TNF-α), surface marker cluster of differentiation 11c/b (CD-11c/b), and ionized calcium-binding adapter molecule 1 (Iba-1), glial fibrillary acidic protein (GFAP). Anti-inflammatory markers include interleukin 10 (IL-10) and transforming growth factor beta (TGF-β). NOD-like receptor family, pyrin domain containing 3 (NLRP3) and apoptosis-associated speck-like protein (ASC), Bcl-2 Associated X protein (BAX), and B-cell Lymphoma 2 (Bcl-2), nicotinamide adenine dinucleotide phosphate (NADPH). Brain-derived neurotrophic factor (BDNF), tropomyosin receptor kinase B (TrkB), glial cell line-derived neurotrophic factor (GDNF) levels, extracellular signal-regulated kinases 1 and 2 (Erk1/2), mitogen-activated protein kinase (MAPK). and cAMP response element-binding protein (CREB). Tyrosine hydroxylase (TH), dopamine transporter (DAT), dopamine level (DA), synaptophysin (Syp), and postsynaptic density protein 95 (PSD-95). 4-hydroxynonenal (4-HNE), nuclear factor erythroid 2-related factor 2 (Nrf2), heme oxygenase-1 (HO-1), NAD(P)H quinone dehydrogenase 1 (NQO1), peroxisome proliferator-activated receptor-gamma coactivator 1-alpha (PGC1-α), nuclear receptor related 1 protein (Nurr1), phosphorylated AMP-activated protein kinase (p-AMPK), glutamate transporter-1 (GLT-1) is elevated, manganese superoxide dismutase (Mn-SOD) and copper-zinc superoxide dismutase (Cu-Zn SOD).

The motor function of the PA group manifested as increased latency and immobility time in 45^o^ inclined grid walking, along with increased akinesia and decreased motor coordination and retention time on the rod in the Rota-rod test. While (PA + Ex) group presented decreased latency and immobility time in inclined grid walking, improved motor coordination, decreased akinesia, and increased retention time on the rod. PA group exhibited decreased distance traveled in the open field test (OFT), impaired equilibrium, and reduced time on the pole test, while the PA + Ex group showed increased distance traveled, improved equilibrium, and increased time on the pole test. Additionally, the PA group displayed increased immobility, reduced balance, walking speed, and stride length in the forced swimming test (FST), and the PA + Ex group demonstrated decreased immobility, improved balance, increased walking speed, and stride length (Table 1).

The PA group exhibited elevated MMP-3, aggregated P-*α*-synuclein, and increased expression of TLR2/4, CD11b/c, surface receptors, and MYD88, a downstream signaling molecule. This PA group also showed increased expression of p/t-IkBα, TRAF6, p/t-TAK1, Iba-1, and GFAP levels. These pathways upregulated P-NF-kB, its subunit P-65, iNOS, and Pro-inflammatory cytokines Pro-IL-1*β*, IL-1β, and TNF-α, while downregulating anti-inflammatory cytokines IL-10, TGF-β in PA group. Only one study presented increased BMX in the striatum. Conversely, the PA + Ex group reduced pGSK3β (Y216) and MMP-3 levels while increasing pGSK3β (S9), preventing α-synuclein aggregation, while a decreased expression of BMX. This PA + Ex group also decreased TLR2/4, CD11b/c, and MYD88 expression, as well as p/t-IkBα, TRAF6, an d p/t-TAK1. PA + Ex also reduced the P-NF-kB and pro-inflammatory cytokines Pro-IL-1β, IL-1β, and TNF-α, alongside an increase in anti-inflammatory cytokines IL-10, TGF-β, and a downregulation of Iba-1 and GFAP. Only a single study reported that PA+ Ex did not affect Iba-1 in substantia nigra (Table 1).

In the apoptotic pathway, the PA group exhibited an increase in the activation of DAMPs, which elevated the expression of cathepsin D, NADPH, and the NLRP3 with ASC inflammasome complex. In the PA group, the increased expression of pro-caspase-1 and its maturation into caspase-1, BAX levels, caspase-3, cleaved caspase-3, and decreased BCL-2 expression were observed. In the PA + Ex group, downregulation was observed in cathepsin D, the NLRP3 with ASC inflammasome complex, NADPH, and the conversion of pro-caspase-1 to caspase-1. The BAX/BCL-2 ratio was reduced, and the expression of caspase-3 and cleaved caspase-3 decreased, inhibiting neuronal apoptosis in the nigrostriatum (Table 1).

BDNF/GDNF-related pathway in PD, the PA group exhibited a consistent decrease in pro-BDNF, BDNF, and the binding receptor TrkB, alongside decreased GDNF levels. The phosphorylation status of Erk1/2 and MAPK-p-Erk1/2 was increased in the PA group. However, the PA + Ex group showed upregulation of pro-BDNF, BDNF, TrkB, and GDNF as well as the downregulation of phosphorylation status, Erk1/2 and MAPK-p-Erk1/2 (Table 1).

In the dopaminergic signaling pathway in PD, the PA group consistently exhibited decreased levels of tyrosine, TH, DAT, dopamine, synaptic proteins synaptophysin, and PSD-95. The PA + Ex group displayed increased expression of TH and DAT proteins and synaptic proteins synaptophysin and PSD-95 (Table 1).

### 3.1 Study quality

The methodological quality of the studies included in this analysis was assessed using the CAMARADES checklist. Scores ranged from 4 to 6 out of 10, indicating moderate methodological quality overall. All studies (100%) were published in peer-reviewed journals and involved animals with induced Parkinson’s disease (PD), accompanied by a statement of compliance with regulatory requirements. Common strengths among the studies included temperature control (63%), randomization of treatment or control (47%), and a statement of conflict of interest (87%). However, only one of the studies mentioned sample size calculation and avoidance of anesthetics with marked intrinsic properties (7%), and none mentioned allocation concealment or blinding (Table 2).

**Table 2:** CAMARADES checklist.

Study	1	2	3	4	5	6	7	8	9	10	Total score
Marino et al. (2023)	✓	✓					✓		✓	✓	5/10
Leem et al. (2023)	✓	✓	✓				✓		✓	✓	6/10
Wang et al. (2022)	✓	✓	✓				✓		✓	✓	6/10
Wang et al. (2021)	✓	✓	✓				✓		✓	✓	6/10
Palasz et al. (2019a)	✓	✓					✓		✓	✓	5/10
Hsueh et al. (2018)	✓	✓					✓		✓	✓	5/10
Real et al. (2017)	✓	✓					✓		✓	✓	5/10
Koo et al. (2017b)	✓	✓					✓		✓	✓	5/10
Jang et al. (2017)	✓	✓	✓				✓		✓		5/10
Churchill et al., 2017	✓		✓				✓		✓	✓	5/10
Shin et al. (2016)	✓	✓	✓				✓		✓		5/10
Hood et al. (2016)	✓	✓					✓		✓	✓	5/10
Toy et al. (2014)	✓		✓				✓		✓	✓	5/10
Tuon et al. (2014)	✓						✓		✓	✓	4/10
Lau et al. (2011)	✓	✓					✓		✓		4/10
Smith et al. (2011)	✓		✓				✓		✓	✓	5/10

(1) Publication in a peer-reviewed journal, (2) Statement of temperature control. (3) Randomization of treatment or control, (4) Allocation concealment, (5) Blinded assessment of outcome, (6) Avoidance of anesthetics with marked intrinsic properties, (7) Use of animals with PD, (8) sample size calculation (9) Statement of compliance with regulatory requirements, (10) Statement regarding possible conflict of interest.

## 4 Discussion

This systematic review presents the first comprehensive analysis of how various exercise training regimens in different animal models of Parkinson’s disease (PD) contribute to nigrostriatal neuronal protection and improve motor function. Diverse exercise training protocols employed in different Parkinson’s models consistently demonstrated improvements in motor coordination, balance, and gait, suggesting a potential role for exercise in ameliorating motor dysfunction. Exercise training reduced nigrostriatal neuronal inflammation across different Parkinson’s models by preventing *α*-synuclein aggregation, inhibiting the TLR/MYD88/IκBα signaling cascade, and decreasing innate immune cells Iba-1 and GFAP, the transcription factor NF-κB, and pro-inflammatory markers TNF-α and IL-1*β*, while increasing anti-inflammatory markers IL-10 and TGF-β. Additionally, exercise training decreased nigrostriatal neuronal apoptosis by downregulating the NLRP3/ASC inflammasome complex, caspase-1, cathepsin D, NADPH, pro-apoptotic BAX, and cleaved caspase-3, while upregulating anti-apoptotic Bcl-2 proteins. The BDNF/GDNF pathway was commonly regulated by exercise training in the nigrostriatum of various Parkinson’s models, providing neurotrophic support by upregulating BDNF and GDNF proteins and downregulating MAPK and p-Erk1/2, thus facilitating CREB activation. Exercise training also upregulated TH and DAT protein expression, dopamine levels, and expression of synaptic proteins such as SYP and PSD-95, which improved the nigrostriatal pathway dopaminergic neurons synaptic activity (Figure 1).

Nigrostriatal neuronal inflammation in Parkinson’s disease (PD), driven by *α*-synuclein aggregation, was reversed by 8 weeks of treadmill training, which also prevented dopamine neuron loss in the substantia nigra and reduced motor impairment in the PFF-α-synuclein PD animal model (Dutta et al., 2022). Subsequently, exercise training led to the downregulation of TLR_2/4_ and downstream signaling molecules such as myeloid differentiation primary response 88 (MyD88), tumor necrosis factor receptor-associated factor 6 (TRAF6), phosphorylated transforming growth factor-b-activated protein kinase 1 (p-TAK1) in the nigrostriatum of difference PD models (Viana et al., 2017; Palasz et al., 2019b). Exercise training was found to increase BMX expression in the striatum of the 6-OHDA-induced PD animal model, which plays a role in regulating the inflammatory balance by inhibiting the phosphorylation of inflammatory signaling proteins (Hsueh et al., 2018; Chen et al., 2004; Chen et al., 2012). Treadmill and strength training for 8 weeks inhibited the Ik*β*a phosphorylation and cytoplasmic protein P65 involved in NF-kB transcription in the striatum of 6-OHDA-induced mice(Tuon et al., 2015). Only one study reported that RWE-induced increases in p-GSK3β (S9) and decreases in p-GSK3β (Y126) in the nigrostriatal system, along with the prevention of *α*-synuclein oligomerization by counteracting MMP-3-induced increases in aggregate-prone species and GSK3β activity-dependent phosphorylation of α-synuclein, led to reduce neuroinflammation(Leem et al., 2023). Furthermore, exercise training led to the downregulation of cell surface markers CD11c/b in innate immune cells, as well as ionized calcium-binding adapter molecule 1 (Iba1) in the nigrostriatum of various animal models (Palasz et al., 2019b; Gil-Martinez et al., 2018), and downregulated GFAP expression in the striatum which prevented the neuronal inflammation in 6-OHDA and MPTP induced PD models (Fallah Mohammadi et al., 2019; Dutra et al., 2012; Vitorino et al., 2022; Al-Jarrah and Jamous, 2011). Results of several studies supported the findings that exercise training in the different animal models MPTP, 6OHDA, and PFF-α-Synuclein of Parkinson’s disease decreased Pro-inflammatory protein TNF-α, and IL-1β expressions by inhibiting the downstream signaling cascade, inhibiting the phosphorylation of NF-kβ and increasing the anti-inflammatory protein IL-10 and TGF-β, which decreased the nigrostriatal neuronal inflammation, further degeneration of dopamine neurons and improved the PD pathology and motor function, balance and gait rhythm (Shih et al., 2015; Leem et al., 2024; Shahidani et al., 2019; Tuon et al., 2015; Leem et al., 2023).

The apoptotic pathway is a critical pathway upregulated in Parkinson’s disease, where damage-associated molecular patterns (DAMPs) increase apoptosis by limiting anti-apoptotic protein Bcl-2 and increasing pro-apoptotic proteins Bcl-xl in the MPTP-induced PD animal model (Chul et al., 2018; Akhtar et al., 2004). Treadmill training for 8 weeks induced neuroprotection of the nigrostriatum against MPTP-induced cell death by suppressing neuronal apoptosis, decreasing BAX expression, and increasing Bcl-2 expression and cleaved caspase-3 in PD animal model (Chul et al., 2018; Koo et al., 2017a). Furthermore, treadmill training 5 days per week for 6 weeks reduced the inflammasome (NLRP3), a large macromolecular complex that contains multiple copies of DAMP receptors, pro-caspase-1, and an adaptor called ASC (apoptotic speck containing protein with a CARD), inhibited the maturation of caspase-1, and inhibited cell death in the substantia nigra of MPTP-induced Parkinson’s mice (Wang et al., 2021). The treadmill training over 4 weeks enhanced autophagy and increased the regeneration of dopamine neurons by limiting pro-apoptotic markers and increasing anti-apoptotic markers. It also enhanced motor performance on the rotarod, motor coordination, distance traveled, stride frequency, reduced stride time, increased walking speed, alleviated dyskinesia, and increased step and stride lengths in pharmacologically induced PD animal models (Chul et al., 2018; Hwang et al., 2018; Minakaki et al., 2019).

The BDNF/GDNF-related pathway, crucially upregulated by exercise training in various Parkinson’s models, provides neurotrophic support to nigrostriatal dopamine neurons, promoting synaptic plasticity and alleviating motor impairment(Palasz et al., 2019b; Tajiri et al., 2010)—fourteen consecutive days of treadmill training reduced the rotational asymmetry and increased the AMP-activated protein kinase, and BDNF expression of in the striatum of 6-OHDA-induced PD animal models (Rezaee et al., 2020; da Costa et al., 2017). The BDNF protein expression increase was consistently observed in the substantia nigra and striatum across various exercise interventions in different PD models including MPTP, 6-OHDA, and PFF in mice and rats (Marino et al., 2023; Palasz et al., 2019a; Hsueh et al., 2018). In contrast, only one study presented increased Pro-BDNF expression in the striatum and hippocampus response to exercise training (Tuon et al., 2014). It was also observed that rotarod walking exercise for 4 weeks and treadmill training for 10 and 18 weeks increased the expression of GDNF in mice with MPTP-induced Parkinson’s model, while evidence supported that 4 weeks of treadmill training increased the striatal GDNF level in the 6-OHDA-induced PD model (Tajiri et al., 2010; Soke et al., 2021). Our study presented uncertainty regarding the impact of exercise training on TrkB expression, with one study presenting upregulation and another indicating no effect (Tuon et al., 2014; Marino et al., 2023). Evidence demonstrated that over four weeks voluntary and forced exercises increased TrkB activation, leading to Erk1/2 phosphorylation and activation of MAPK downstream signaling pathways, as well as CREB gene expression in the nigrostriatum of different animal models (Soke et al., 2021; Wu et al., 2011; Zhou et al., 2024). Exercise training increased neurotrophic support to dopamine neurons, increased synaptic activity in the striatum, and improved motor skills and anti-dyskinetic responses in PD animal models (Speck et al., 2019; Almikhlafi, 2023).

**Figure 2:**
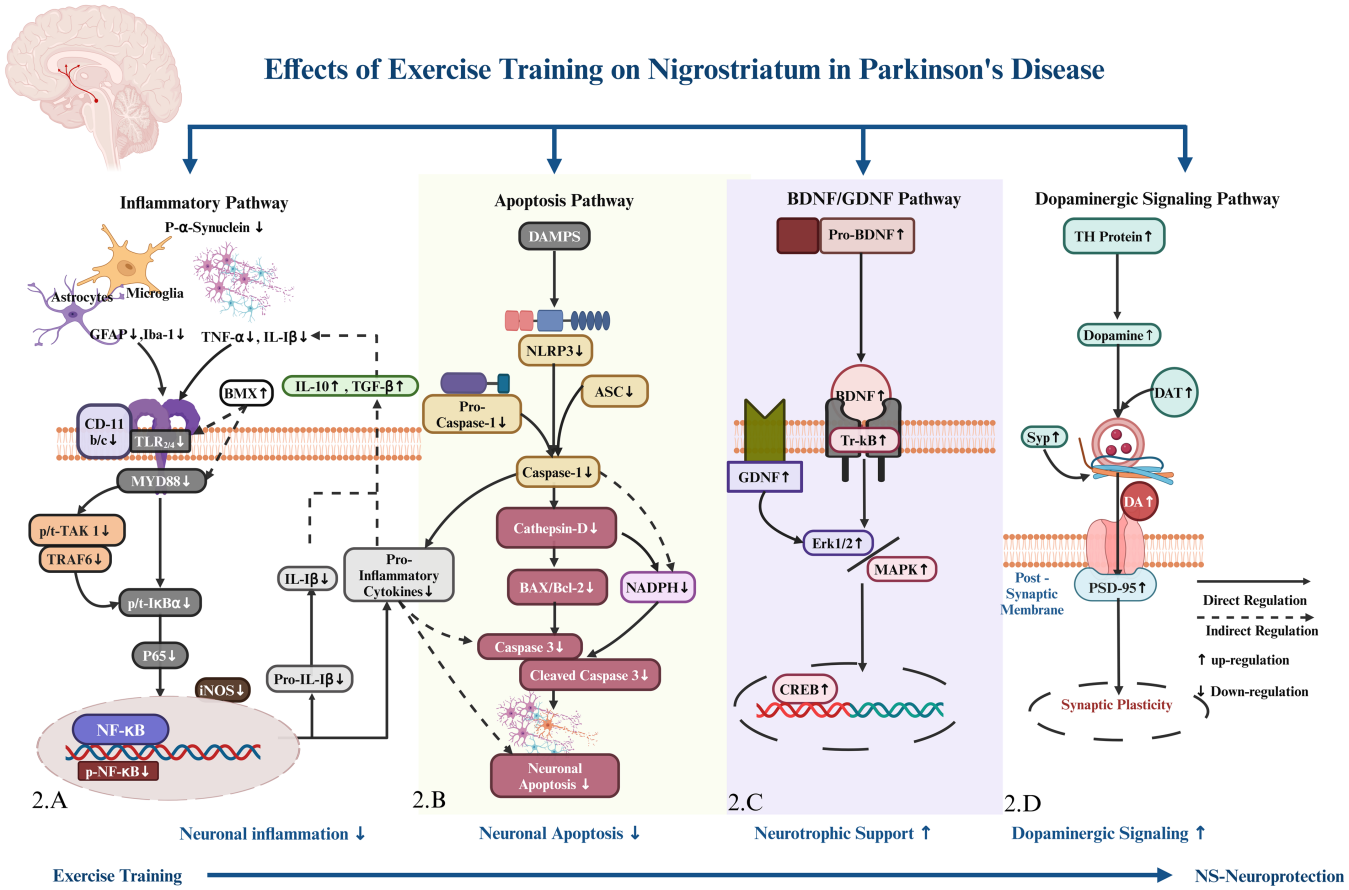
Hypothetical pathway regulated by exercise training for nigrostriatal (NS) neuronal protection. **(A)** Toll-like receptor (TLR) & binding protein Myeloid Differentiation Primary Response 88 (MYD88), Bone Marrow Tyrosine Kinase in Chromosome X (BMX) protein, Tumor Necrosis Factor Receptor Associated Factor 6 (TRAF6), TGF-beta Activated Kinase 1 (TAK-1), an Inhibitor of Kappa B alpha (IκB*α*), Protein 65 (P 65), a subunit of, Nuclear Factor kappa B (NF-kB), inducible Nitric Oxide Synthase (iNOS), Interleukin 1 Beta (IL-1*β*), Tumor Necrosis Factor-alpha (TNF-α), surface marker Cluster of Differentiation 11c/b (CD-11c/b), and Ionized Calcium-Binding Adapter Molecule 1 (Iba-1), Glial Fibrillary Acidic Protein (GFAP). Anti-inflammatory markers include Interleukin 10 (IL-10) and Transforming Growth Factor beta (TGF-β). **(B)** NOD-like receptor family, pyrin domain containing 3 (NLRP3) and Apoptosis-associated Speck-Like Protein (ASC), Nicotinamide Adenine Dinucleotide Phosphate (NADPH). Bcl-2 Associated X Protein (BAX), and B-cell Lymphoma 2 (Bcl-2). **(C)** Brain-Derived Neurotrophic Factor (BDNF), Tropomyosin Receptor Kinase B (TrkB), Glial Cell Line-Derived Neurotrophic Factor (GDNF), Extracellular Signal-Regulated Kinases 1 and 2 (Erk1/2), Mitogen-Activated Protein Kinase (MAPK), and cAMP Response Element-Binding Protein (CREB). Tyrosine Hydroxylase (TH), **(D)** Dopamine Transporter (DAT), Dopamine Level (DA), Dopamine Receptors (DARs), Synaptophysin (Syp), and Postsynaptic Density Protein 95 (PSD-95),

The dopaminergic signaling pathway was upregulated through exercise training in different animal strains of Parkinson’s disease, which increased striatal tyrosine hydroxylase (TH) protein expression and dopamine (DA) levels (Minakaki et al., 2019; Rezaee et al., 2020; Tsai et al., 2019; Koo et al., 2017a; Dutta et al., 2022; Fallah Mohammadi et al., 2019; Goes et al., 2014). Fourteen days of treadmill training increased the expression of the dopamine transporter (DAT), a transmembrane protein responsible for DA reuptake from the synaptic cleft to increase dopamine levels, in the striatum of rats with 6-OHDA-induced PD animal model (da Costa et al., 2017). Moreover, treadmill training for 5 days per week for 4 and 6 weeks increased the synaptic protein expressions of synaptophysin (Syp) and PSD-95 in the striatum of MPTP-induced animals, which increased synaptic integrity and dendritic spine length (Shin et al., 2016; Takamatsu et al., 2010; Toy et al., 2014). Exercise training prevented the loss of dopaminergic neurons. It increased the synaptic plasticity in the nigrostriatum of different pharmacologically induced PD animal models, which improved motor function, gait parameters, and balance (Almikhlafi, 2023; Lai et al., 2019; Chen et al., 2018; Sekar et al., 2018; Palasz et al., 2019b) (see Figure 2).

### 4.1 Limitations

Our systematic review (SR) presents several limitations. This study focused solely on proteomics regulation by exercise training within the nigrostriatum, excluding other brain regions such as the motor cortex, hypothalamus, and ventral tegmental area. The nigrostriatum is a crucial brain region involved in motor performance and is primarily implicated in Parkinson’s disease. This precise focus may give more appropriate findings about the disease perspective in this brain location. Additionally, the study summarized only motor functions or motor behaviors of animals, omitting non-motor functions. The restriction to only three databases—PubMed, EMBASE, and Web of Science—may have led to the omission of relevant studies available elsewhere. The male representation in multiple studies (*n* = 15) and the limited inclusion of female animals (*n* = 1) raise concerns about the generalizability of the findings. Most studies included in this systematic review inducted young animals of 8–12 weeks may limit the applicability of results in different age populations. The effect of exercise training on the hypothalamus, motor cortex, pituitary axis, and sympathetic nervous system should be addressed in future studies. Hemiparkinsonian models may impact the evaluation of motor function; therefore, only bilateral Parkinson’s models were included.

## 5 Conclusion

This systematic review detailed how exercise training influenced protein expressions in the nigrostriatal pathway. It highlighted its role in reducing neuronal inflammation and apoptosis while enhancing BDNF/GDNF-related and dopaminergic signaling pathways in the nigrostriatum of Parkinson’s disease animal models. Exercise training mitigated neuronal inflammation by decreasing the accumulation of *α*-synuclein, inhibiting the downstream signaling cascade and phosphorylation of NF-kB, reducing pro-inflammatory cytokines IL-1β and TNFα, and increasing anti-inflammatory cytokines IL-10 and TGFβ. It also reduced apoptosis by downregulating the NLRP3 inflammasome complex, the BAX/BCL-2 ratio, and caspase 3. Exercise training upregulated BDNF and GDNF protein expression, providing neurotrophic support to dopamine neurons. Additionally, it enhanced nigrostriatal dopaminergic signaling by increasing the expression of dopaminergic proteins such as TH, DAT, and dopamine, as well as synaptic proteins like synaptophysin and PSD-95, thereby improving structural and functional connectivity. Overall, these findings suggested that exercise training contributed to protecting nigrostriatal neurons and improved motor function by enhancing motor coordination, balance, and gait.

## Data Availability

The original contributions presented in the study are included in the article/supplementary material, further inquiries can be directed to the corresponding authors.
